# Effect of Ambient pH on Growth, Pathogenicity, and Patulin Production of *Penicillium expansum*

**DOI:** 10.3390/toxins13080550

**Published:** 2021-08-07

**Authors:** Carelle Kouasseu Jimdjio, Huali Xue, Yang Bi, Mina Nan, Lan Li, Rui Zhang, Qili Liu, Lumei Pu

**Affiliations:** 1 College of Science, Gansu Agricultural University, Lanzhou 730070, China; jimdjiocarelle@gmail.com (C.K.J.); nanmn@gsau.edu.cn (M.N.); ahh127316@outlook.com (L.L.); liuqili2018@outlook.com (Q.L.); pulm@gsau.edu.cn (L.P.); 2 College of Food Science and Engineering, Gansu Agricultural University, Lanzhou 730070, China; zhangrui12111020@outlook.com

**Keywords:** *Penicillium expansum*, patulin, gene expression, ambient pH, pathogenicity

## Abstract

*Penicillium expansum* is an important postharvest pathogen of pomaceous fruit and a causal agent of blue mold or soft rot. In this study, we investigated the effect of ambient pH on growth, ultrastructure alteration, and pathogenicity of *P. expansum*, as well as accumulation of patulin and expression of genes involved in patulin biosynthesis. Under different pH, the fungus was routinely cultured and collected for growth, pathogenicity, patulin production, and gene expression studies using transmission electron microscopy, apple inoculation, HPLC, and RT-qPCR methods. Different ambient pH had significant impact on expression of genes and growth factors involved in patulin biosynthesis. Under same range of pH, gene expression profile, growth factors, and patulin accumulation (in vivo and in vitro) all showed similar changing trends. A well-developed cell was observed in addition to upregulation of genes at pH between pH 5.0 and 7.0, while the opposite was observed when pH was too basic (8.5) or too acid (2.5). Additionally, ambient pH had direct or indirect influence on expression of *PecreaA, PelaeA*, and *PepacC*. These findings will help in understanding the effect of ambient pH on growth, pathogenicity, and patulin production and support the development of successful methods for combating *P. expansum* infection on apple fruits.

## 1. Introduction

*Penicillium expansum* is a ubiquitous, opportunistic, and saprophytic fungus belonging to the Ascomycete sub-group and *Trichomaceae* family. It is one of the most important postharvest pathogens of pomaceous fruits (e.g., apples and pears) and a causal agent of blue mold or soft rot [1]. The fungus attacks fruits through injuries or mechanical wounds during postharvest handling and storage, leading to blue mold on fruits. The blue mold not only causes substantial economic loss around the world but also leads to patulin contamination with public health consequences [2]. Patulin may lead to nausea, vomiting, gastrointestinal injuries, kidney damage, cancerous tumor growth, genetic mutations, and embryonic developmental defects. Patulin belongs to a shortlist of mycotoxins (including aflatoxins, ochratoxin A, zearalenone, fumonisins, and trichothecenes) found in foods and their contamination levels are controlled in many countries around the world. European Regulation 1881/2006 has set a maximum standard of 50 μg/L for fruit juices and derived products, 25 μg/L for solid apple products, and 10 μg/L for juices and foods intended for infants and young infants [3]. Therefore, inhibition of growth and patulin production of *P. expansum* is essential in maintaining the security of products derived from apples. 

Environmental factors, including temperature, pH, gas composition, water activity, and other chemical characteristics of substrates influence growth, pathogenicity, and patulin production of *P. expansum*. Among these factors, ambient pH plays an essential role. Manteau et al. [4] reported that pH might dictate living cells’ survival, particularly on microorganisms, since their cells are in direct contact with the environment. Zong et al. [5] also suggested that ambient pH significantly influences growth, sporulation, and biomass of *P. expansum*, as well as patulin production; Damoglou et al. [6] reported that pH strongly influences patulin production and stability. It has also been reported that *P. expansum* acidifies its host by stimulating glucose oxidase 2 (gox2) and catalyzes glucose oxidation to secrete D-gluconic acid (GLA) which can modulate ambient pH, thereby activating several polygalacturonases that act in favor of successful fungal colonization [7,8]. The fungus then secretes pathogenic factors and metabolites such as patulin. 

pH, the main environmental factor of this study, has significant influence on patulin production. For many cellular activities, such as development, morphogenesis, cell membrane and cell wall stabilization, protein stability and function, secondary metabolism, and host infection, ambient pH usually acts as essential signal [9]. Thus, pH acts on genes that code for patulin biosynthesis and affects gene expression of metabolic growth factors. According to Chen et al. [10], ambient pH may influence spore germination of *P. expansum* by modifying intracellular pH, protein expression, and regulatory factors. Additionally, it has been reported that genes that code for permease enzymes and export metabolites are affected by environmental pH [11]. For instance, in the case of *Aspergillus nidulans* (filamentous fungi), *pacC* gene codes for *pacC* transcription factor that functions in alkaline conditions as an activator of genes and in acidic conditions as a repressor of expressed genes and possibly involve in homeostasis of internal pH [12]. In addition, Chen et al. [10] found that mutant *PepacC* lost the ability to generate patulin at pH values above 6.0 because expression of all genes in the patulin biosynthesis pathway gene cluster were substantially downregulated. 

Other regulatory proteins such as *laeA* (global secondary metabolism transcription factors) and *creA* (carbon metabolism inhibition transcription factors) are known to affect natural product biosynthesis in fungi. The gene cluster, which facilitate patulin biosynthesis, has been identified and characterized [13,14,15]. A number of studies have reported the effect of ambient pH on patulin biosynthesis; however, there are few systematic studies on the effect of ambient pH on growth, ultrastructure alteration, pathogenicity, patulin production, and regulation of *P. expansum*. Therefore, the present study aimed at investigating the effect of ambient pH on growth, ultrastructure alteration, and pathogenicity of *P. expansum* as well as accumulation and regulation of patulin (in vivo and in vitro), and the expressions of transcription factors (*pacC, creaA, and laeA*) involved in patulin biosynthesis.

## 2. Results

### 2.1. Effect of Ambient pH on P. expansum Growth In Vitro

#### 2.1.1. Effect of Different Ambient pH on Spore Germination and Germ Tube Length of *P. expansum*

**Figure 1:**
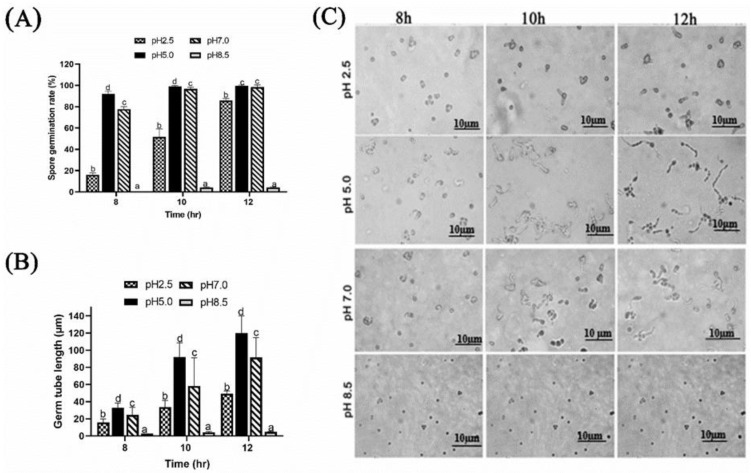
Effect of ambient pH on the growth of *P. expansum.* (**A**) Spore germination, (**B**,**C**): Germ tube length. The lower-case letters indicate significant difference at *p* < 0.05 at each point.

The effect of pH on growth factors was established by culturing *P. expansum* in potato dextrose broth (PDB) modified at various pH and incubated at different periods. The pH value of 5.0 was most beneficial for *P. expansum* spore germination and germ tube growth. After 8 h, germination rate at pH of 5.0 and 7.0 were 95% and 75%, respectively, while germination rate at pH of 2.5 was around 15%, and that of pH 8.5 was insignificant compared with pH 5.0 and 7.0. After 12 h, germination rate reached more than 90% at pH 5.0 and pH 7.0, while at pH 2.5, it was about 70% and remained non-existent at pH 8.5 (Figure 1A). The average germ tube length was around 110 µm after 12 h, compared to a considerable reduction in germ tube length at exposure to pH of 2.5 and 8.5 (Figure 1B,C). 

#### 2.1.2. Effect of Different Ambient pH on Colony Diameter and Colony Edges of *P. expansum*

**Figure 2:**
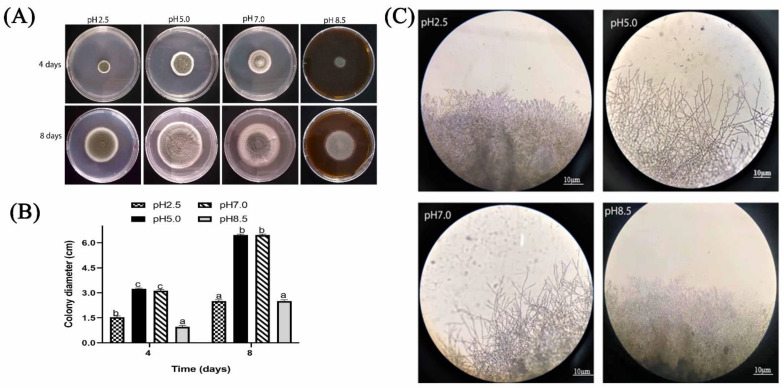
Effect of different ambient pH on *P. expansum*. (**A**) Colony diameter of *P. expansum* for 8 days at 25 °C on PDA adjusted at different pH. (**B**) Statistical data for *P. expansum* colony diameter. (**C**) Colony edges were observed under light microscope. The lower-case letters indicate significant difference at *p* < 0.05 at each point.

The results (Figure 2A,B) showed that colony diameters at pH 5.0 and 7.0 were significantly higher than that at pH 2.5 and 8.5 (*p* < 0.05). For instance, the diameter of the colony at pH 5.0 enlarged to 6.4 cm after 8 days of culture on potato dextrose agar (PDA) medium, and the colonies looked greenish, velvet, and powdery. At pH 2.5 and 8.5, the colonies had less powdery appearance, and their color was not very pronounced; their diameter was approximately equal to 2.5 cm. The colony edges at pH 5.0 and 7.0 were more apparent than that at pH 2.5 and 8.5, and the branches were longer, while the edges were short and dense at pH 2.5 and 8.5. The edges were not pronounced at pH 8.5 (Figure 2C). 

#### 2.1.3. Effect of Ambient pH on Ultrastructural Alteration of *P. expansum*

**Figure 3:**
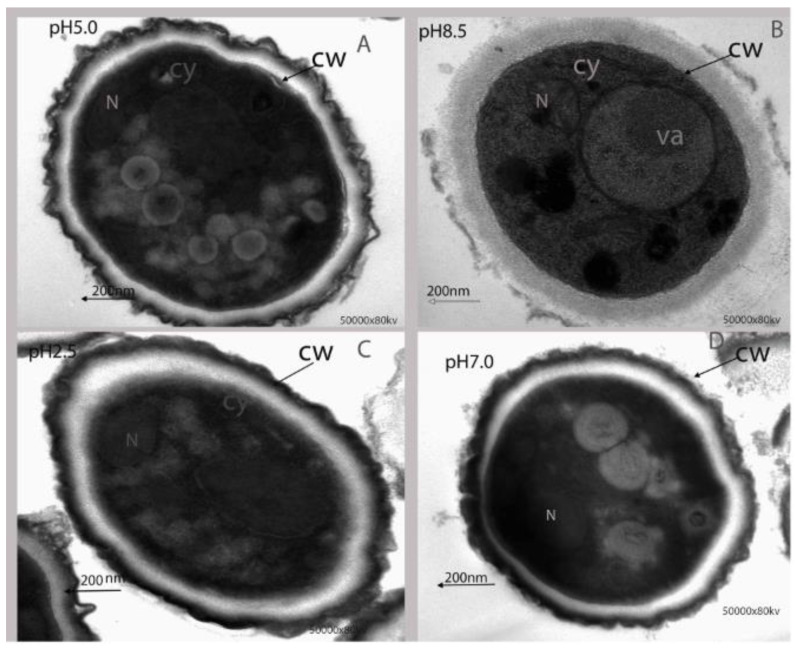
TEM observation of *P. expansum* under different ambient pH treatments. This was repeated three times, and at least two blocks were detected for each replicate. Va, vacuole; CW, cell wall; Cy, cytoplasm; N, nucleus. (**A**) pH = 5.0, (**B**) pH = 8.5, (**C**) pH = 2.5, (**D**) pH = 7.0.

Transmission electron microscopy (TEM) analysis was carried out to further investigate ultrastructural changes of *P. expansum* under different ambient pH. The results indicated that cell ultrastructure was normal at pH 5.0, with intact cell membrane and normal organelles (Figure 3A). In contrast, at pH 8.5 the cell wall was strongly destroyed, vacuole expanded, and plasma was not homogenous (Figure 3B). Additionally, at pH 2.5 and pH 7.0, the cell wall was slightly damaged, and organelles were disorganized (Figure 3C,D).

#### 2.1.4. Effect of Different Ambient pH on Sporulation, Biomass, and Patulin Production of *P. expansum*

**Figure 4:**
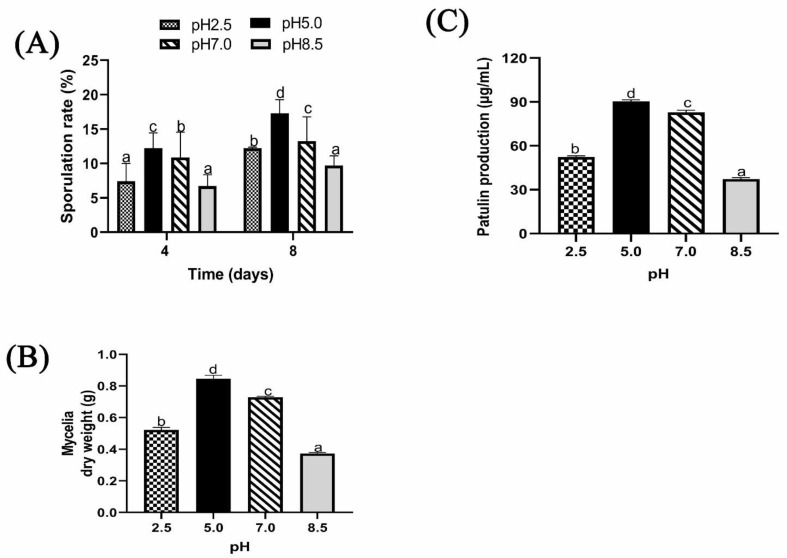
Effect of ambient pH on sporulation rate, mycelia biomass, and patulin production. (**A**) Sporulation rate; (**B**) Mycelia dry weight; (**C**) Patulin production. The lower-case letters indicate significant difference at *p* < 0.05 at each point.

The effect of different ambient pH on sporulation of *P. expansum* in PDB is shown in Figure 4A. After 4 days of culture, sporulation was greatly enhanced at pH 5.0 followed by pH 7.0 with significant difference. The sporulation at pH 5.0 was approximately 2-fold more than that at pH 8.5 and 1.4-fold more than that at pH 2.5. After 8 days of culture, the same sporulation trend was observed. 

Mycelia of *P. expansum* were cultured for 8 days in PDB culture modified at pH of 2.5, 5.0, 7.0, and 8.5 to illustrate the impact of ambient pH on mycelia growth and patulin production. The highest biomass was found at pH 5.0, followed by pH 7.0, pH 2.5, and pH 8.5 (Figure 4B). Patulin production displayed similar biomass trend. The concentration of patulin significantly decreased when pH value was less than 5.0 or more than 7.0 (Figure 4C). The concentration of patulin was 90.4 µg mL^−1^ and 82.9µg mL^−1^ at pH 5.0 and 7.0, respectively, whereas it was found to decrease at pH 2.5 and pH 8.5, with the concentration of 52.3 µg mL ^−1^ and 37.3 µg mL^−1^, respectively.

#### 2.1.5. Effect of Ambient pH on Relative Expression of Genes Encoding Transcription Factors and Proteins Involved in Patulin Biosynthesis

**Figure 5:**
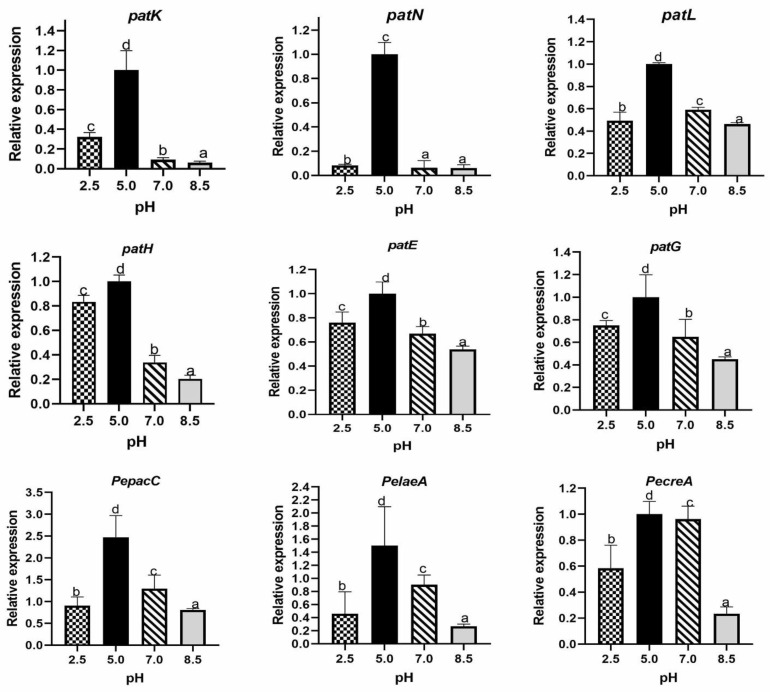
Expression of genes potentially involved in patulin biosynthesis of *P. expansum* on media buffered at different ambient pH. Data were analyzed by the 2^−ΔΔCt^ method, and normalized using β-tubulin housekeeping gene. The lower-case letters indicate significant difference at *p* < 0.05 at each point.

There are about 55 gene clusters potentially relating to secondary metabolism and includes a cluster of 15 genes that is involved in patulin biosynthesis of *P. expansum* [13,14,15]. In this study, *PepatE*, *PepatN*, *PepatL*, *PepatK*, *PepatG* and *PepatH* were analyzed under different ambient pH conditions using RT-qPCR. The results showed enormous expression of genes at pH 5.0 followed by pH 7.0. It was also observed that *PepatK* and *PepatH* were less expressed at pH 7.0 and 8.5 than other genes expressed at the same pH; the different ambient pH showed significant effect on expression of investigated transcription factor genes. *PepacC* expression was very high at pH 5.0; similarly, high expression of *PecreA* and *PelaeA* was also observed at pH 5.0 (Figure 5). 

### 2.2. Effect of Ambient pH on Pathogenicity Induced by P. expansum and Patulin Production In Vivo 

**Figure 6:**
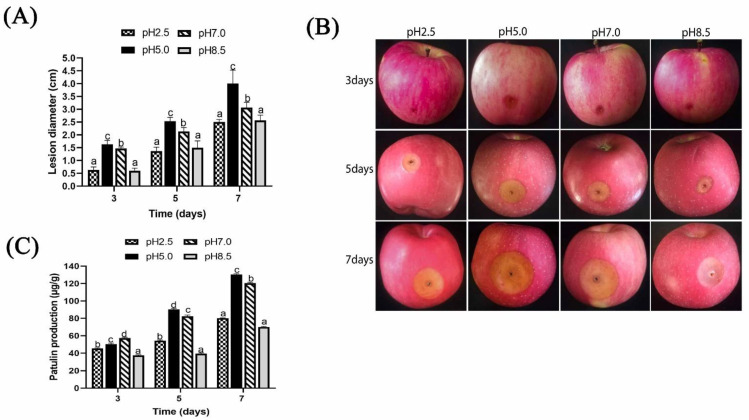
Effect of ambient pH on pathogenicity induced by *P. expansum* in apples; (**A**) Lesion diameter; (**B**) Disease symptoms on apple fruits; (**C**) Patulin production using rotten apple parts. Lower case letters indicate significant difference at *p* < 0.05 at each point.

Under different ambient pH, *P. expansum* was highly pathogenic to apple fruits at pH 5.0 and pH 7.0 (Figure 6 A,B). At 7 days, the result displayed an expanded lesion diameter measuring 4.5 cm at pH 5.0, followed by that of pH 7.0, which was 3.0 cm, while at pH 2.5 and pH 8.5, the lesion diameter was around 2.5 cm. 

The concentration of patulin significantly increased with time of infection (Figure 6C). For the first three days, patulin production was highest at pH 7 (57 ug/g) fresh weight, then after 5 and 7 days, the highest patulin production was recorded at pH 5.0. For instance, at 7 days, the patulin inoculated apple fruits were 130 µg/g, thus was 1.62-fold and 1.86-fold higher than those of pH 2.5 and pH 8.5.

## 3. Discussion

According to literature, *P. expansum* is among the most ubiquitous fungi in apple fruits. Infected fruits and derived products are unwholesome to human health due to patulin contamination [16,17]. To effectively control mycotoxin contamination, it would be judicious to investigate environmental factors that affect *P. expansum* growth, patulin production, and expression of patulin biosynthesis associated genes.

In the present study, effect of different ambient pH on *P. expansum* growth and patulin synthesis (in vivo and in vitro) were studied. Furthermore, expressions of 6 genes involved in patulin biosynthesis, as well as 3 transcription factors (metabolic growth factors) were examined under same ambient pH. We found that, at pH 2.5 and 8.5, spore germination and germ tube length were significantly inhibited, whereas germination improved at pH 5.0 (Figure 1A–C). It has been reported that ambient pH can affect spore germination, secretome profile, and pathogenicity of plant fungal pathogens [18,19].

In addition, it was observed that growth factors of *P. expansum* markedly decreased under pH 2.5 and pH 8.5. After 7 days, colony diameter of *P. expansum* increased significantly at pH 5.0 and 7.0, but decreased significantly at pH 8.5 and 2.5 (Figure 2A,B). The effect of ambient pH on PDA diameter was found to have positive relationship with biomass. Biomass significantly increased in PDB medium at pH 5.0 and 7.0 compared to 2.5 and 8.5. The reason could be that strong acids or bases destroyed chromosomes and proteins’ DNA, thus inhibiting conidia growth. Tannous et al. [20] developed a mathematical model that facilitated estimation of *P. expansum* growth rate under temperature, water activity (AW), and pH. At 24 °C with pH of 5.1 and high Aw level of 0.99 W, an optimal growth rate of 0.92 cm per day was predicted [20]. Using TEM analysis, the structure of spores at different ambient pH was better understood. Spores under pH 5.0 were well-organized with homogenous cytoplasm, entire cell walls, and typical organelles. In contrast, at pH 7.0, 2.5, and 8.5, some damages were observed. At pH 8.5, the cells’ vacuole enlarged, indicating potential osmotic stress on *P. expansum* conidia caused by high basic environment; additionally, the cell wall was greatly destroyed (Figure 3B). Furthermore, apples inoculated with *P. expansum* spores adapted to different ambient pH and demonstrated reduced disease incidence at pH 2.5 and 8.5, but a higher disease incidence was observed at pH 5.0 and 7.0 (Figure 4A,B). The result showed that large amounts of patulin were produced (in vivo and in vitro) under acidic conditions (pH 2.5 and 5.0) more than under alkaline conditions (pH 8.5) (Figure 4C and Figure 6C). Earlier, it was demonstrated that patulin was more stable at a pH range of 2.5 to 5.5 than in a more basic pH [20,21]. In addition, a similar report on other mycotoxins such as aflatoxin, sterigmatocystin, and norsoloronic acid showed that their levels increased 5- to 10-fold in cultures grown at pH 5.0 or 4.0 compared to pH 8.0 [22]. Moreover, ochratoxin has been confirmed to be constant at pH ≤ 4 and significantly higher than those observed at pH ≥ 5 [23]. In many fungi, secondary metabolism is considered to be closely associated with sporulation [24,25]. In this study, it was found that patulin production (in vivo and in vitro) correlated with sporulation rate under different ambient pH. However, Calvo et al. [26] speculated that aflatoxin and sterigmatocystin production appears to be influenced by the growth medium’s pH. Studies on the effect of pH on these mycotoxins have shown complex and contradictory results. At pH 4.0 or below, in *A. flavus*, sclerotial production reduced by 50%, while aflatoxins production was maximum. Simultaneously, it was shown that *A. nidulans* and *A. parasiticus* produced less mycotoxin as pH of growth media increased. 

The results also revealed a significant link between *PepatL* gene expression and other genes (*PepatE, PepatN, PepatK, PepatG, and PepatH*) at the same range of pH. This result is not surprising because earlier, *PepatL* was presented as a global transcription factor and its disruption led to reduced patulin production with sharp decrease in gene expression [27,28]. These genes (*PepatE, PepatN, PepatK, PepatG, and PepatH*) play crucial role in patulin biosynthesis. Their up-regulation at favorable pH (pH 5.0) directly correlates with patulin production. For example, *PepatK* codes for 6-methyl salicylic acid synthase (6MSAS) which is involved in the synthesis of 6-methyl salicylic acid at the first step of patulin biosynthesis, and *PepatN* codes for isoepoxydon dehydrogenase which catalyzes one of the last steps in the pathway [29]. This result can be supported by Ballester et al. [15], who demonstrated the inability of *PEX2* strain of *P. expansum* to produce patulin due to significant down-expression of *PepatK* gene and progressive decrease in expression of *PepatN* and *PepatL* genes after the first days of inoculation.

Global transcription factors can be activators or repressors for genes facilitating secondary metabolite production depending on many environmental conditions. This result showed an up-regulation of *PepacC, PecreA,* and *PelaeA* in vivo at pH 5.0 (Figure 5). *PepacC,* previously described as pH-dependent transcription factor for patulin production, is an activator in acidic conditions but a repressor in an alkaline condition and required for mycelia growth and conidiation virulence in *P. expansum* [10]. It also plays a role in physiological functions such as growth, differentiation, and pathogenicity in several yeast and fungi through transcriptional activation and inhibition of genes [30]. In *F. graminearum*, the same trend was observed where all *tri* genes displayed rapid repression in response to neutralization concomitants with *Fgpac1* induction [31]. The deletion mutant *Fg∆Pac1* exhibited slower development in neutral and alkaline pH, enhanced sensitivity to H_2_O_2_, and facilitated earlier *tri* gene induction and toxin accumulation in acidic pH [31]. On the contrary, in *A. nidulans* and *P. chrysogenum*, *pacC* transcript levels were higher in alkaline than acidic growth conditions [32,33]. Previously it was reported that modification of pH from 2.5 to 3.5 increased patulin accumulation by 10-fold and *PelaeA* by 1.9-fold [34]. Loss of *creA* had profound effect on growth, sporulation, and germination of *P. expansum*. *creA* has been shown to express during biosynthesis of patulin, which is significantly affected by carbon source [35,36]. The results thus suggest that unfavorable conditions (pH 2.5 and 8.5) might decrease the ability of *P. expansum* to produce proteolytic enzyme or impaired the CCR pathway, which is driven by *creA* in filamentous fungi [36]. Our findings are consistent with a previous study which reported that, *PecreA* regulation of patulin production is independent of *PelaeA* [37].

## 4. Conclusions

In conclusion, the findings of this study revealed that various ambient pH has significant impact on growth factors and genes involved in patulin biosynthesis. Under the same range of pH, growth factors, gene expression profile, and patulin production (in vivo and in vitro) all showed similar results. A well-developed cell was observed in addition to upregulation of genes at pH between pH 5.0 and 7.0, while the opposite results were observed when pH was too basic (8.5) or too acid (2.5). Our results also showed that ambient pH has influence on expression of *PecreaA*, *PelaeA,* and *PepacC*. These findings will help to understand the effect of ambient pH on patulin production and support the development of successful methods for combating *P. expansum* attacks on apple crops.

## 5. Materials and Methods

### 5.1. Apple Fruits

Apple fruit (*Malus Domestica*) of the Fuji cultivar was purchased from Tiaoshan Farm in Jingtai County of Gansu Province in China, transported to the laboratory, and kept at ambient temperature (7–10 °C, relative humidity (RH) 70–80%). The fruits had uniform size without any infection or mechanical injuries. The apple fruits were washed twice with running water from tap, then surface-sterilized by immersing in 2% NaClO for 2 min, washed with sterile distilled water, and finally dried by air according to the method described by Sanzani et al. [38].

### 5.2. Fungal Strain and Basal Medium Preparation

*Penicillium. expansum* T01 was provided by the Institute of Botany, Chinese Academy of Sciences. The fungus was routinely cultured on potato dextrose agar (PDA) plate for 7 days at 25 °C. Subsequently, the culture was collected to prepare spore suspension using sterile distilled water and 0.05% (*v/v*) Tween 80. The spore suspension was filtered with four layers of sterile cheesecloth, and concentration of the spore suspension was adjusted to 1 × 10^6^ spore mL^−1^ according to the method of Qin et al. [39]. Potato dextrose broth (PDB) medium was prepared and buffered with 0.2 M Na_2_HPO_4_·12H_2_O and 0.1 M C_6_H_8_O·7H_2_O while the pH was adjusted to 2.5, 5.0, 7.0, and 8.5, respectively, by using pH meter.

### 5.3. Studies on the Effect of Ambient pH on Growth Factors of P. expansum In Vitro

#### 5.3.1. Studies on the Effect of Ambient pH on Spore Germination and Germ Tube Elongation of *P. expansum*

Aliquots of 1 mL of spore suspension were added to 100 mL pH-adjusted PDB media in 250 mL conical flasks to a final concentration of 1 × 10^6^ spore mL^−1^ and cultured at 25 °C on a rotary shaker at 200 rpm. After incubation for 8, 10, and 12 h, the germination rate of approximately 100 spores was calculated microscopically for each treatment. Then, the length of the germ tube of 65 spores per treatment was measured at the same period. Three replicates were performed for each treatment, and the experiment was repeated twice.

#### 5.3.2. Studies on the Effect of Ambient pH on Colony Diameter and Colony Edges *of P. expansum*

To illustrate the effect of pH on colony diameter of *P. expansum,* agar powder was added to 250 mL of buffered PDB media (3.5% was added to the PDB media buffered at pH 2.5 while 2% of agar powder was added to the PDB media buffered at pH 5.0, 7.0 and 8.5) in order to obtain a solid medium (PDA). After sterilization, the PDA was poured into Petri dishes, and then 5 µL of spore suspension (1 × 10^6^ spore mL^−1^) was added onto the center of the solidified PDA plates and cultured for 8 days at 25 °C [9]. The colony diameter was measured each day for 8 days, and colony edges were observed under light microscope. Each experiment was performed in triplicates and repeated three times.

#### 5.3.3. Studies on the Effect of Ambient pH on Ultrastructural Alteration of *P. expansum*

After 7 days of culturing *P. expansum* on PDA at 25 °C, conidia were harvested and suspended in PDB media adjusted at different pH in 250 mL flask to a final concentration of 10^7^ spore mL^−1^. After incubation at 25 °C at 120 rpm for 7 days, in a rotary shaker, mycelia were obtained by filtering, and then fixed with 2% glutaraldehyde, followed by washing, dehydrating, through different solutions of acetone-ethanol, and finally incorporated in epoxy medium. The mycelia were observed and photographic images were captured under transmission electron microscope (TEM H-7650 Hitachi, Tokyo, Japan). Each experiment was performed in triplicates and repeated three times. 

#### 5.3.4. Studies on the Effect of Ambient pH on Sporulation, Mycelia Biomass and Patulin Production

Determination of sporulation was according to the method of Zhen et al. [9] with slight modification. *P. expansum* was cultured on pH-adjusted PDA for 4 and 8 days, and 40 mL of distilled water with 0.05 % (*v/v*) tween 80 was used to wash the Petri dishes, and then transferred into 50 mL tubes. To get conidiophores from conidia, the tubes were oscillated at the speed of 600 oscillations per min. Haemocytometer was used to determine sporulation. Each experiment was performed in triplicates and repeated three times. 

The mycelia biomass of *P. expansum* was evaluated in PDB medium at different pH values after 8 days. Mycelium was cultured in a pH-adjusted PDB medium, then filtered through whatman paper, and dried in an oven at 60 °C. Subsequently, mycelia dry weight was determined using analytical balance. At the same time, the supernatant was filtered through a 0.45 µm filter for patulin content determination. High-performance chromatography analysis (HPLC) method was used with slight modification of the mobile phase [40] for patulin content determination. Then, 10 μL of the extract was injected into a liquid chromatography machine (Waters Corp., Milford, MA, USA) equipped with an autosampler (Waters 2707), binary HPLC pump (Waters 1525), and a UV/Visible Detector (Waters 2487) using a C18 column (5 μm, 250 × 4.6 mm, Intersil ODS-3, GL Sciences, Tokyo, Japan). The modified mobile phase consisted of a mixture of water and acetonitrile (90: 10, *v/v*) at 1 mL/min flow rate in isocratic elution mode with 276 nm detection wavelength.

#### 5.3.5. Studies on the Effect of Ambient pH on Expression of Genes Involved in Patulin Biosynthesis In Vitro 

##### Primer Design and Total RNA Extraction 

Primers for initial screening of *PepatE, PepatN, PepatL, PepatK, PepatG, PepatH, PecreA, PelaeA, PepacC*, and *β-tubulin* genes were designed with premier primer 6 software and synthesized. According to the manufacturer’s instructions, total RNA was extracted from 0.1 g aliquots of mycelia sample using Trizol reagent (Tiangen Biotech, Beijing, China). Briefly, Trizol reagent was added to minced tissues (0.1 g) and incubated on ice for 5 min to obtain RNA pellets. Reverse transcription of RNA into cDNA was performed using standard reverse-transcription polymerase chain reaction (RT-PCR) protocol from PrimeScript RT reagent Kit plus gDNA Eraser (Perfect Real Time, TaKaRa, Dalian, China). The synthesized cDNA was stored at −80 °C for RT-qPCR experiments. Detailed information on RT-qPCR primers is presented in Table 1.

##### RT-qPCR and Relative mRNA Expression Analysis 

The mRNA expression levels of *PepatE, PepatN, PepatL, patK, patG, patH, PecreA, PelaeA,* and *PepacC* were compared between pH of 2.5, 5.0, 7.0, and 8.5. All primers showed similar PCR efficiency. Total cDNA in a 20 μL reaction volume was analyzed in an ABI Step One Plus Real-Time PCR System (Applied Biosystems, Foster City, CA, USA) using SYBR Premix Ex Taq TM (Tli RNaseH Plus) quantitative fluorescence kit (TaKaRa, Dalian, China) and 10 μM of reverse and forward primers each. The reaction volume also contained 10 μL of SYBR Premix Ex TaqTM, 0.4 μL of each primer, 0.4 μL of ROX Reference Dye, 2 μL of cDNA, and 6.8 μL of RNase-free water. PCR reaction was first performed at 95 °C for 3 min, and then followed by 40 cycles at 95 °C for 7 s, subsequently 55 °C for 10 s; finally, 72 °C for 15 s. β-tubulin was used as an internal control to normalize the amount and quality of each cDNA. The relative expression of target genes was calculated by the 2^−ΔΔCt^ method [41]. All RT-qPCRs were run in triplicates and repeated three times. 

#### 5.3.6. Studies on the Effect of Ambient pH on Pathogenicity and Patulin Accumulation in Apple Fruits Inoculated with *P. expansum* In Vivo

The experiment was performed according to methods described by Xue et al. [42] with slight modification. Three (3) groups of apple fruits each containing 60 apples were inoculated with *P. expansum*; After sterilization of apple fruits, holes (2 mm in diameter and 6 mm in depth) were created aseptically on each fruit’s equator by using sterilized pipette tips, each hole was inoculated with 5 µL of spore suspension (1 × 10^5^) prepared with distilled water adjusted at different pH levels. The inoculated fruits were kept on a clean and sterilized plastic box (25 ± 2 °C; RH 70–75%). In order to maintain the pH value at inoculation site, from start of inoculation to the end of sampling time, a sterile 0.2 M Na_2_HPO_4_·12H_2_O and 0.1 M C_6_H_8_O·7H_2_O buffer solution at pH 2.5, pH 5.0, pH 7, and pH 8.5 was injected into the inoculation wells at 12 h intervals. The lesion diameter was measured after inoculation at 3, 5, and 7 days. Subsequently, the rotten tissues were separately excised from each fruit and immediately stored at −80 °C for patulin analysis. The extraction and determination of patulin were performed according to Zong et al. [5]. The experiment was performed in triplicates, with 180 fruits for each replication.

### 5.4. Statistical Analysis

Statistical analysis was carried out using SPSS version 17.0 (SPSS, Inc., Chicago, IL, USA). The data were analyzed with one-way analysis of variance (ANOVA) and Duncan’s multiple range test. Data were expressed as standard error (SE) of the mean and differences at *p* < 0.05 were considered statistically significant.

## Figures and Tables

**Table 1:** Information on RT-qPCR primers.

Gene	Primer Sequences (5’-3’)
*PepatE*	F: CATTCTCATCGGGCCTGAGT
	R: TCGAAGCTCTTCCGGACATG
*PepatN*	F: CGTTCGATGTCGCTAGCAAA
	R: GGCGATAATCACGTCAATTCG
*PepatL*	F: GCAGGAGATCCGTTTCAGACA
	R: CCACTGACCGACGGTTACAAC
*PepatK*	F: GACGCTGGGCTACTGGATTG
	R: TCGTGCGTGAGGCCAGTAT
*PepatG*	F: CGGCCGTCTTGAAGGAAAT
	R: CTTGCCGTAGCGGGTGAATA
*PepatH*	F: CATTTATCGGCGGTGTTCTGA
	R: GATCAACGCTTGCACGATAGC
*PecreaA*	F: CGATACTTCGCCCGACTCA
	R: TGAGAGGCGGTAGCAAGCTAA
*PelaeA*	F: CCCGAGAAATACCCGAATCA
	R: TCACACGGAAGCGGGTAGAT
*PepacC*	F: TGAGGCTGGTACTGCCGAAT
	R: CAACTCCTTCCATGGCATCA
*β-tubulin*	F: CTCCAGCTCGAGCGTATGAA
	R: GGCTCCAAATCGACGAGAAC

## Data Availability

The datasets used and/or analyzed during the current study are available from the corresponding author upon reasonable request.
